# Matching Participants for Triceps Surae Muscle Strength and Tendon Stiffness Does Not Eliminate Age-Related Differences in Mechanical Power Output During Jumping

**DOI:** 10.3389/fphys.2018.01345

**Published:** 2018-09-25

**Authors:** Matthias König, Svenja Hemmers, Gaspar Epro, Christopher McCrum, Thijs Maria Anne Ackermans, Ulrich Hartmann, Kiros Karamanidis

**Affiliations:** ^1^School of Applied Sciences, Sport and Exercise Science Research Centre, London South Bank University, London, United Kingdom; ^2^Department of Mathematics and Technology, RheinAhrCampus Remagen, Koblenz University of Applied Sciences, Remagen, Germany; ^3^NUTRIM School of Nutrition and Translational Research in Metabolism, Department of Nutrition and Movement Sciences, Maastricht University Medical Centre, Maastricht, Netherlands; ^4^Institute of Movement and Sport Gerontology, German Sport University Cologne, Cologne, Germany; ^5^School of Sport and Exercise Sciences, Liverpool John Moores University, Liverpool, United Kingdom

**Keywords:** leg stiffness, mechanical power, jumping, muscle strength, tendon stiffness, aging, motor control

## Abstract

Reductions in muscular power output and performance during multi-joint motor tasks with aging have often been associated with muscle weakness. This study aimed to examine if matching younger and middle-aged adults for triceps surae (TS) muscle strength and tendon stiffness eliminates age-related differences in muscular power production during drop jump. The maximal ankle plantar flexion moment and gastrocnemius medialis tendon stiffness of 29 middle-aged (40–67 years) and 26 younger (18–30 years) healthy physically active male adults were assessed during isometric voluntary ankle plantar flexion contractions using simultaneous dynamometry and ultrasonography. The elongation of the tendon during the loading phase was assessed by digitizing the myotendinous junction of the gastrocnemius medialis muscle. Eight younger (23 ± 3 years) and eight middle-aged (54 ± 7 years) adults from the larger subject pool were matched for TS muscle strength and tendon stiffness (plantar flexion moment young: 3.1 ± 0.4 Nm/kg; middle-aged: 3.2 ± 0.5 Nm/kg; tendon stiffness: 553 ± 97 vs. 572 ± 100 N/mm) and then performed series of drop jumps from different box heights (13, 23, 33, and 39 cm) onto a force plate (sampling frequency 1000 Hz). The matched young and middle-aged adults showed similar drop jump heights for all conditions (from lowest to highest box height: 18.0 ± 3.7 vs. 19.7 ± 4.8 cm; 22.6 ± 4.2 vs. 22.9 ± 4.9 cm; 24.8 ± 3.8 vs. 23.5 ± 4.9 cm; 25.2 ± 6.2 vs. 22.7 ± 5.0 cm). However, middle-aged adults showed longer ground contact times (on average 36%), lower vertical ground reaction forces (36%) and hence lower average mechanical power (from lowest to highest box height: 2266 ± 563 vs. 1498 ± 545 W; 3563 ± 774 vs. 2222 ± 320 W; 4360 ± 658 vs. 2475 ± 528 W; 5008 ± 919 vs. 3034 ± 435 W) independent of box height. Further, leg stiffness was lower (48%) in middle-aged compared to younger adults for all jumping conditions and we found significant correlations between average mechanical power and leg stiffness (0.70 ≤ *r* ≤ 0.83; *p* < 0.01). Thus, while jumping performance appears to be unaffected when leg extensor muscle strength and tendon stiffness are maintained, the reduced muscular power output during lower limb multi-joint tasks seen with aging may be due to age-related changes in motor task execution strategy rather than due to muscle weakness.

## Introduction

Lower limb muscular power production is crucial for an effective and safe locomotion in sport and during activities of daily living. For example, rapid muscle force generation is required during various daily lower limb multi-joint tasks and has been identified as a predictor for mobility (Rantanen and Avela, 1997; Suzuki et al., 2001; Bean et al., 2002; Cuoco et al., 2004) and falls risk (Skelton et al., 2002) in older adults.

Previous research suggests an age-related decline in muscular power production of the leg extensor muscles during single (e.g., maximal isokinetic ankle plantar flexion contractions; Thom et al., 2005, 2007) and multi-joint tasks such as walking (DeVita and Hortobagyi, 2000), running (Karamanidis et al., 2006; DeVita et al., 2016) and jumping (Ferretti et al., 1994; Wang, 2008) and that this decline has already begun by middle age (Kulmala et al., 2014). As joint mechanical power is the product of torque and angular velocity, one might suggest that this decrease in power generation capacity might be caused by age-related reductions in muscle strength (Asmussen and Heebøll-Nielsen, 1962; Viitasalo et al., 1985; Häkkinen and Häkkinen, 1991; Lindle et al., 1997) and muscle fiber maximal shortening velocity (Larsson et al., 1997; Korhonen et al., 2006). Furthermore, it is well established that the mechanical properties of the tendon affect the force potential of the muscle due to the force–length–velocity relationship (Hof et al., 2002; Roberts, 2016). For instance, higher patellar tendon stiffness has been shown to facilitate the rate of torque development during isometric knee extension contractions (Reeves et al., 2003; Bojsen-Møller et al., 2005). Therefore, the age-related decrease in leg extensor muscle-tendon unit (MTU) mechanical properties (i.e., isometric muscle strength and tendon stiffness; Karamanidis and Arampatzis, 2005, 2006; Onambele et al., 2006; Mademli and Arampatzis, 2008; Stenroth et al., 2012) may be a major contributor to the reduced muscular power production during lower limb multi-joint tasks seen with aging. Changes in lower limb muscular power production during running previously have been associated with an age-related deterioration in leg extensor isometric muscle strength and tendon stiffness (Karamanidis et al., 2006). However, it is yet not clear if the age-related differences in muscular power output during lower limb multi-joint tasks can solely be explained by changes in leg extensor muscle strength and tendon stiffness seen with aging.

A common approach for testing muscular power production of the leg extensor muscles during lower limb multi-joint tasks is maximal vertical jumping (Bojsen-Møller et al., 2005; Holsgaard Larsen et al., 2007; Caserotti et al., 2008; Edwén et al., 2014). Muscular power output during maximal vertical jumping (i.e., maximal counter-movement jumps and drop jumps; DJs) has previously been associated with the stiffness of the lower limb joints (referred to as ankle and knee joint stiffness or leg stiffness; Arampatzis et al., 2001a,b; Korff et al., 2009). When controlling for leg stiffness during maximal drop jumps (dropping from a box, and upon contact with the ground, executing a maximal vertical jump) by influencing ground contact times through verbal instructions, it has been shown that a maximization of mechanical power is achieved by optimal leg stiffness values (Arampatzis et al., 2001a,b). In an aging context, there is evidence that ankle joint stiffness during the braking phase of maximal DJs on a sledge apparatus is reduced with aging, causing a diminished jumping performance in older compared to younger adults (Hoffrén et al., 2007). However, as most studies comparing young and older adults included older adults with lower MTU mechanical properties, it remains unclear to what extent leg extensor isometric muscle strength and tendon stiffness or other neuromuscular factors (e.g., leg stiffness) contribute to the age-related changes in muscular power and performance output during lower limb multi-joint tasks. One might overcome this drawback by matching different age groups for one or more investigated parameters. For instance, analyzing younger and older adults with similar leg extensor muscle strength causes a reduction in the age-related differences in joint kinetics during gait, but these differences still remain significant, indicating that factors other than leg extensor muscle strength mediate the age-related changes in gait mechanics (Hortobágyi et al., 2016).

In the current study, we aimed to investigate if age-related differences in DJ performance (jumping height) and kinetics (maximal vertical ground reaction force, ground contact time, average mechanical power, and leg stiffness) would be eliminated when young and middle-aged adults are matched for triceps surae (TS) isometric muscle strength and tendon stiffness, in order to test the hypothesis that age-related differences in muscular power production during lower limb multi-joint tasks cannot solely be explained by alterations in leg-extensor muscle strength and tendon stiffness seen with aging. Therefore, the second aim of this study was to determine to what extent leg stiffness (referred to as the ratio of maximal vertical ground reaction force and the maximum vertical displacement of the body’s center of mass during ground contact) is associated with the average mechanical power output during maximal vertical jumping in young and middle-aged adults with the hypothesis that leg stiffness may be a major factor of the age-related decline in muscular power production during lower limb multi-joint tasks. As it has been shown that the mechanical properties of the TS MTU in particular are crucial for lower limb multi-joint tasks (e.g., walking, running, sprinting and jumping; Hof et al., 2002; Lichtwark and Wilson, 2007; Pandy and Andriacchi, 2010; Kulmala et al., 2014; Huang et al., 2015; Farris et al., 2016), the DJ task was chosen due to the specific requirements of TS muscle force generation. Further, by using different box heights we aimed to examine whether the outcome measures (and the potential age effects on these measures) vary with changes in task demand.

## Materials and Methods

### Participants

Twenty-nine middle-aged (40–67 years) and 26 younger (18–30 years) physically active healthy male adults gave their written informed consent to participate in this study. Participants were included when two participants, one from the young and one from the middle-aged groups had similar TS MTU mechanical properties. Finally, eight middle-aged (range: 41–67 years) and eight younger (19–28 years) adults were matched for TS muscle strength and tendon stiffness. None of the participants had experience in regularly performing DJs. Exclusion criteria were any previous Achilles tendon ruptures, Achilles tendon injuries (e.g., tendinopathy) or pain within the last 12 months, or musculoskeletal impairments in the lower limbs (e.g., ankle joint pain), which could have influenced the outcomes of this investigation. The study was approved by the university’s human ethics committee and was conducted according to the Declaration of Helsinki.

### Analysis of Triceps Surae Muscle Strength and Tendon Stiffness

The experimental setup to assess TS MTU mechanical properties has been described previously in detail (Ackermans et al., 2016). Briefly, participants were seated on a custom-made strain gauge-type dynamometer (1000 Hz; TEMULAB, Protendon GmbH & Co. KG, Aachen, Germany) with their lower leg secured and the knee and ankle joints positioned at 90 degrees (foot and thigh perpendicular to the shank) and the foot placed on the dynamometer foot plate. The measurements began with a regimented warm-up of ten submaximal contractions guided by TEMULAB software and three maximal isometric contractions to precondition the tendon (Maganaris, 2003). Following this, the TS muscle strength and tendon force-elongation properties of the dominant leg were assessed during a maximal isometric voluntary ramp contraction (MVC) and three subsequent sustained contractions at 30, 50, and 80% of the determined maximal joint moment (Ackermans et al., 2016; McCrum et al., 2018b) by integrating dynamometry and ultrasonography (27 Hz; MyLab^TM^One, Esaote, Genua, Italy). All sustained contractions were guided by visual feedback displayed on a computer screen showing the produced joint moment. In the current study, force values of ±5% of the target force were accepted (when the force was held for 3 s). If this was not achieved, the specific trial was repeated. During all maximal contractions, strong verbal encouragement was given to the participants in order to attain their actual MVC (McNair et al., 1996). By aligning the axis of rotation of the ankle and the force plate’s center of rotation on the dynamometer, the ankle joint plantar flexion moment could be considered equal to the moment of the force plate (Ackermans et al., 2016). The gravitational forces were taken into account for all participants before each contraction. Achilles tendon force was calculated by dividing the resultant ankle joint plantar flexion moment by the tendon moment arm obtained from the literature (Maganaris et al., 1998). Please note that in the current model the magnitude of Achilles tendon force was estimated by calculating the resultant ankle joint moment via inverse dynamics without considering muscle activation and, therefore, we could not account for the moment contributions of all the other agonistic ankle plantar flexor muscles or antagonistic dorsiflexors. The gastrocnemius medialis (GM) tendon was examined using an ultrasound probe securely fixed on the GM within a rigid custom-made casing. Elongation of the GM tendon during the loading phase was determined by manually tracking the GM myotendinous junction in custom-made Matlab software (Matlab 2013b, MathWorks Inc., Natick, MA, United States). The effect of inevitable ankle joint angular rotation on the measured tendon elongation during contractions (Magnusson et al., 2001) was taken into account by multiplying the tendon moment arm by the ankle joint angular changes during contraction. In the present study, we used a potentiometer located under the heel, measuring any heel lift during loading and calculated the ankle joint angle changes via the inverse tangent of the ratio of the heel lift to the distance between the head of the fifth metatarsal bone and the potentiometer. This method was found to be in accordance with the results obtained using a motion capture system with an average difference in ankle joint angle changes of less than 1.1 degrees during maximal isometric plantar flexion contractions (Ackermans et al., 2016). Tendon stiffness was then subsequently determined as the ratio of the increase in the calculated tendon force and the increase in elongation from 30 to 80% of maximal tendon force. However, it is mandatory to address the fact that we did not account for the relative contribution of the GM to Achilles tendon force affecting our calculated GM tendon stiffness in absolute terms.

### Analysis of Drop Jump Kinetics

On a second occasion, the TS muscle strength and stiffness-matched participants performed a series of three DJs from each different box height (13, 23, 33, and 39 cm; DJ13–DJ39, respectively) onto a force plate (90 cm × 60 cm, 1000 Hz; Kistler, Winterthur, Switzerland) in a randomized order. In order to avoid any effects through muscle fatigue, about 90 s of rest was provided between each trial. Since ground contact time influences leg stiffness during maximal vertical jumping (Arampatzis et al., 2001a,b), participants were instructed to jump as high as possible with as short contact time as possible. Prior to the measurements, all participants performed three familiarization sessions of the DJ task. Jumping height was determined using the flight time method (Asmussen and Bonde-Petersen, 1974) and ground contact time (defined as the time interval from the first instant when vertical ground reaction force reached a threshold value of 20 N to the first instant below 20 N) and maximal vertical ground reaction force during the support phase were determined. The average mechanical power was calculated by dividing the total work performed during the jump (summed work during the negative and the positive dynamic phase) by the ground contact time. Leg stiffness was assessed using the spring-mass model (Blickhan, 1989) and calculated as:

$$KLeg=\frac{F_{max}}{\Delta CoM}$$

where *F_max_* is the maximal vertical ground reaction force and *ΔCoM* is the maximum vertical displacement of the body’s center of mass during ground contact (Hobara et al., 2008). Vertical center of mass displacement was obtained by double integration of the vertical acceleration with respect to time:

$$CoM(t)=\iint \frac{F(t)-mg}{m}dt\;dt$$

where *F* represents the vertical ground reaction force, *m* is the body mass and *g* is the gravitational acceleration (Hobara et al., 2008). As lower limb stiffness has been shown to scale with body mass (Granata et al., 2002), leg stiffness was normalized to body mass and expressed as kN/m/kg.

### Statistics

For all participants, the trial with the greatest jumping height for each jumping condition (box height) was taken into account for further analysis. A two-way repeated measures analysis of variance (ANOVA; factors: age group and box height) was performed in order to detect potential effects of age or box height on DJ height or kinetics with respect to average mechanical power, leg stiffness, maximal vertical ground reaction force and ground contact time. Detected significant main effects or interactions were further analyzed using Duncan’s *post hoc* comparison. Anthropometric data, age, maximal isometric ankle joint plantar flexion moments and GM tendon stiffness were compared with *t*-tests for non-dependent samples. In order to determine the relationship between average mechanical power and leg stiffness during DJs from different box heights, Pearson’s correlation coefficients were computed. All statistical analyses were performed using the software Statistica (Release 10.0, Statsoft; Tulsa, OK, United States). The level of statistical significance was set at α = 0.05. All results provided in the text, tables and figures are presented as mean and standard deviation (SD).

## Results

**FIGURE 1:**
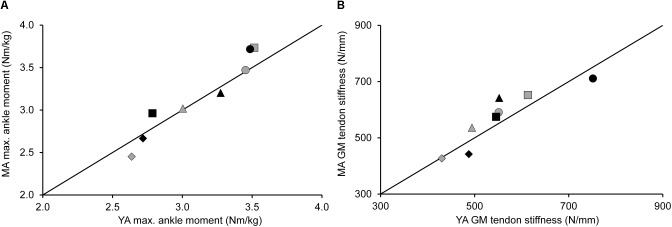
Maximal ankle joint plantar flexion moment **(A)** and gastrocnemius medialis (GM) tendon stiffness **(B)** for the TS muscle strength and stiffness-matched young (YA; *n* = 8) and middle-aged (MA; *n* = 8) adults. Different symbols represent the participant pairs. The data points remain close to the identity line for both parameters, indicating that there were no statistically significant differences in maximal ankle joint plantar flexion moment and GM tendon stiffness between groups. Maximal ankle joint plantar flexion moment was normalized to body mass.

**FIGURE 2:**
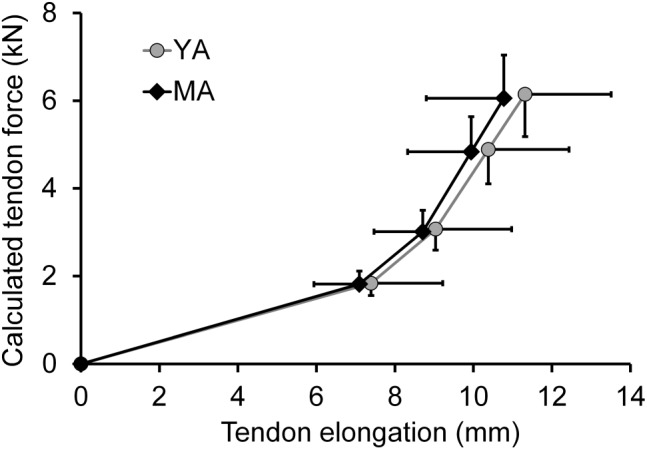
Force-elongation relationship (mean and SD) of the gastrocnemius medialis tendon for the TS muscle strength and stiffness-matched young (YA; *n* = 8) and middle-aged (MA; *n* = 8) adults. Data points are displayed at 0, 30, 50, 80, and 100% of maximal calculated tendon force.

The eight young and eight middle-aged adults were matched for TS isometric muscle strength and tendon stiffness (see **Figures 1, 2**), which meant that there were no significant differences in maximal isometric ankle joint plantar flexion moments and GM tendon stiffness between young (3.1 ± 0.4 Nm/kg and 553 ± 97 N/mm, respectively) and middle-aged adults (3.2 ± 0.5 Nm/kg and 572 ± 100 N/mm). Paired samples *t*-tests revealed a significant group difference in age (23 ± 3 vs. 54 ± 7 years; *p* < 0.001), but no significant differences in body height (178.3 ± 6.5 cm vs. 179.9 ± 4.7 cm) and mass (79.3 ± 9.6 kg vs. 75.3 ± 4.9 kg).

**FIGURE 3:**
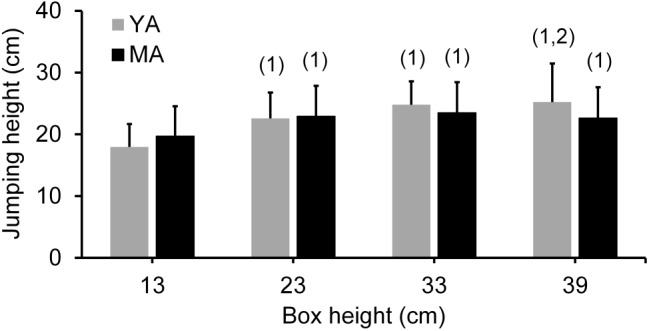
Jumping performance during maximal drop jumps from four box heights in TS muscle strength and stiffness-matched young (YA; *n* = 8) and middle-aged (MA; *n* = 8) adults. There was no statistically significant age difference in drop jump height, independent of jumping condition. ^1^Statistically significant (*p* < 0.05) difference to 13 cm; ^2^Statistically significant (*p* < 0.05) difference to 23 cm.

Concerning the DJ performance, the implemented two-way ANOVA revealed a statistically significant age group × box height interaction for jumping height [*F*(3,42) = 3.04, *p* < 0.05]. Duncan’s *post hoc* analysis revealed no differences in jumping height between TS muscle strength and stiffness-matched young and middle-aged adults independent of jumping condition (see **Figure 3**; Cohen’s *d* over all box heights = 0.08 ≤ *d* ≤ 0.46). Jumping height was significantly lower for the 13 cm compared to all other box heights in both age groups. However, a lower DJ performance for the 23 cm compared to the 39 cm condition was only found in younger adults.

**FIGURE 4:**
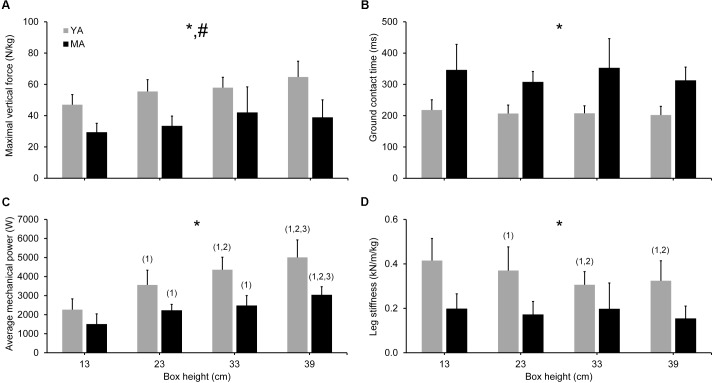
Jumping kinetics during maximal drop jumps from four box heights in TS muscle strength and stiffness-matched young (YA; *n* = 8) and middle-aged (MA; *n* = 8) adults. There were statistically significant age differences in maximal vertical ground reaction force **(A)**, ground contact time **(B)**, average mechanical power **(C),** and leg stiffness **(D)**, independent of box height. Maximal vertical ground reaction force and leg stiffness were normalized to body mass. ^∗^Statistically significant (*p* < 0.05) difference between YA and MA; ^#^statistically significant (*p* < 0.001) box height effect; ^1^statistically significant (*p* < 0.05) difference to 13 cm; ^2^statistically significant (*p* < 0.05) difference to 23 cm; ^3^statistically significant (*p* < 0.001) difference to 33 cm.

Considering the DJ kinetics, there were significant age effects on ground contact time [*F*(1,14) = 31.39, *p* < 0.001; 2.04 ≤ *d* ≤ 3.30] and maximal vertical ground reaction force during support phase [*F*(1,14) = 26.35, *p* < 0.001; 1.27 ≤ *d* ≤ 3.17], with the middle-aged adults showing longer ground contact times and lower forces for all box heights (no interaction effect; see **Figure 4**). The two-way ANOVA revealed a statistically significant age × box height interaction for average mechanical power [*F*(3,42) = 18.21, *p* < 0.001]. Duncan’s *post hoc* analysis revealed significant differences in average mechanical power between age groups independent of box height (1.39 ≤ *d* ≤ 3.16) and higher power values with increasing box height (see **Figure 4**). However, the middle-aged adults showed no significant difference in average mechanical power for the 23 and 33 cm conditions. Further, there was a statistically significant age group × box height interaction for leg stiffness [*F*(3,42) = 4.76, *p* < 0.01]. Duncan’s *post hoc* analysis revealed lower leg stiffness values in the middle-aged compared to younger adults for all jumping conditions (1.18 ≤ *d* ≤ 2.57) and lower leg stiffness values with raising box height only in the younger adults (see **Figure 4**). However, no significant difference in leg stiffness was found between the 33 and 39 cm conditions.

**FIGURE 5:**
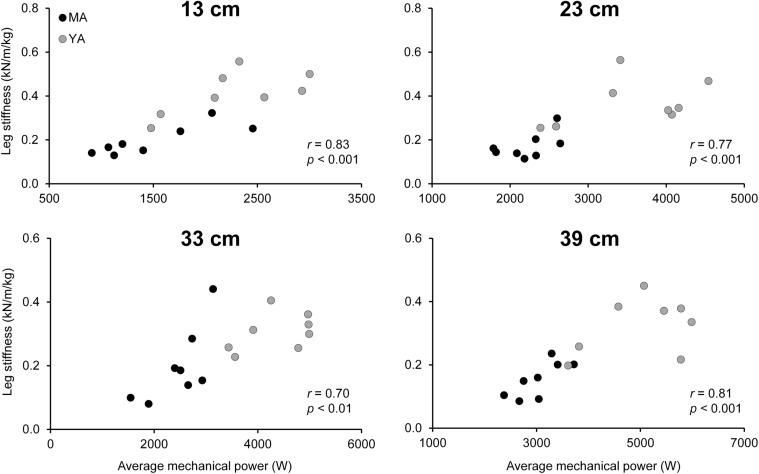
Relationship between average mechanical power and leg stiffness during maximal drop jumps from four box heights in TS muscle strength and stiffness-matched young (YA; *n* = 8) and middle-aged (MA; *n* = 8) adults. Leg stiffness was normalized to body mass.

Regarding a potential box height effect on DJ kinetics, there was a significant (*p* < 0.001) effect of jumping condition on maximal vertical ground reaction force during support phase [*F*(3,42) = 17.23], with higher vertical ground reaction forces with increments in box height. However, no significant difference in maximal vertical ground reaction force could be detected for the 33 and 39 cm conditions. Significant positive correlations were found between average mechanical power and leg stiffness over all analyzed participants (*n* = 16; 0.70 ≤ *r* ≤ 0.83; *p* < 0.01; see **Figure 5**).

## Discussion

The first aim of this study was to investigate if age-related differences in DJ performance and muscular power generation would be eliminated when young and middle-aged adults are matched for TS isometric muscle strength and tendon stiffness in order to test the hypothesis that age-related differences in muscular power production during lower limb multi-joint tasks cannot solely be explained by alterations in leg-extensor muscle strength and tendon stiffness seen with aging. In agreement with our hypothesis, middle-aged compared to younger adults generated lower average mechanical power during DJs despite being matched for TS isometric muscle strength and tendon stiffness. Our results are robust across four different jumping conditions i.e., task demands. Thus, they reflect previous findings seen in walking, showing that age-related differences in joint kinetics remained significant when matching younger and older adults for leg extensor muscle strength (Hortobágyi et al., 2016).

There might be potential factors other than maximum isometric muscle strength and tendon stiffness contributing to the age-related differences in leg extensor muscle power production during lower limb multi-joint tasks. Accordingly, the second aim of this study was to analyze whether a diminished muscular power production during maximal vertical jumping may be associated with potential differences in leg stiffness between age groups. In the current study, we found remarkably lower leg stiffness values in the middle-aged adults and significant positive correlations between leg stiffness and average mechanical power during support phase of maximal DJs from different box heights (0.70 ≤ *r* ≤ 0.83; *p* < 0.01), meaning that approximately 50 to 70% of the variability in average mechanical power during support phase can be related to the variance in leg stiffness within the pooled group of middle-aged and younger adults. These results support previous findings showing a significant relationship between muscular power output and the stiffness of the lower limb joints during maximal vertical jumping (Arampatzis et al., 2001a,b; Korff et al., 2009), indicating that leg stiffness is a major contributor to muscular power production during lower limb multi-joint tasks. Previously, aging has been associated with a less efficient utilization of tendon elasticity during maximal DJs on a sledge apparatus (Hoffrén et al., 2007). In the current study, TS MTU mechanical properties (maximal isometric muscle strength and tendon stiffness) were matched between younger and middle-aged adults and hence, we found no significant age effect on GM tendon maximal energy storage capacity (i.e., integral under the GM tendon force-elongation-relationship during the loading phase of a MVC; young 215 ± 67 J; middle: 199 ± 80 J). Thus, one might speculate that the observed lower leg stiffness values in combination with the lower maximal vertical ground reaction forces in the middle-aged compared to younger adults may have led to a reduced amount of elastic strain energy stored in the GM tendon during the negative phase of the DJ potentially affecting power generation during the positive phase.

In the literature several factors have been associated with leg stiffness during lower limb multi-joint tasks. For example, leg stiffness is influenced by the stiffness of the passive elastic structures and the ability to appropriately activate the agonistic and antagonistic muscles (Hortobágyi and DeVita, 2000; Hoffrén et al., 2007) in order to stiffen the lower limb joints. During maximal DJs on a sledge apparatus, both a lower activation of the plantar flexor muscles and higher antagonistic coactivity during the braking phase have been related to a lower ankle joint stiffness experienced in older compared to younger adults (Hoffrén et al., 2007). Since TS isometric muscle strength and tendon stiffness were matched in the current study for middle-aged and younger adults, the lower leg stiffness in the middle-aged adults may be possibly explained by age-specific muscle activation patterns. However, with the current experimental set up (i.e., no electromyographic recordings of the lower limb muscles were taken) it cannot be directly answered whether possible age-related changes in muscle activation may be the primary drivers for the observed differences in leg stiffness during jumping between young and middle-aged adults or if other additional factors (e.g., differences in TS MTU parallel elastic structures) might also play a role.

Despite the above mentioned differences in muscular power production and leg stiffness between the two age groups, matching younger and middle-aged adults for TS isometric muscle strength and tendon stiffness resulted in no age-related differences in DJ height, independent of jumping condition (i.e., box height), indicating that leg-extensor isometric muscle strength and tendon stiffness play an important role for the performance in explosive motor tasks (e.g., DJ). The current finding, that a similar performance during maximal vertical jumping can be achieved by different motor task execution strategies i.e., levels of leg stiffness is in accordance to previous results (Arampatzis et al., 2001a,b). In the current study, middle-aged adults showed lower maximal vertical ground reaction forces and longer ground contact times compared to younger adults, suggesting that middle-aged adults maximize their jumping height by prolonging the time over which joint moments at the lower extremities are applied to the ground, due to a larger compliance of their lower limb joints during support phase. This is in line with earlier findings, showing lower maximal ground reaction forces but longer push-off phases in older compared to younger adults during hopping (Hoffrén et al., 2011) or DJs on a sledge apparatus (Hoffrén et al., 2007). Although not significant, there was a continuous increase in ground reaction force from 13 to 39 cm for the younger (with the exception of the 23 vs. 33 cm conditions) but not for the middle-aged adults (no differences between the 13 vs. 23, 23 vs. 39 and 33 vs. 39 cm conditions). These results suggest that middle-aged adults adopt a motor task execution strategy keeping maximal vertical ground reaction forces and hence, impact loads, on the musculoskeletal system within critical limits by less stiffening of their lower limb joints during the support phase compared to younger adults. This is supported by the reduced leg stiffness values with raising box height (i.e., task demand) even in younger adults. However, based on the current findings we cannot exclude that the middle-aged adults were not able to stiffen their lower limb joints to the same amount as younger adults, due to deficits in leg extensor muscle activation, for example (Stackhouse et al., 2001; Stevens et al., 2001; Morse et al., 2004; Clark et al., 2013).

A limitation of the current study may be that we did not use optical motion capture analysis but used a more convenient approach in order to assess mechanical power during maximal DJs. However, average mechanical power was calculated by the ratio of total work and ground contact time for all age groups and, therefore, we believe that this drawback potentially affects our results more in absolute terms than the validity of the corresponding comparative data. Regarding our ankle-knee joint configuration for GM tendon stiffness assessment, the contribution of the GM muscle to the net joint moment may be reduced compared to a fully extended knee joint angle since the GM operates on the ascending limb of the force-length relationship. However, in a previous study (Ackermans et al., 2016) we found no significant differences in tendon mechanical properties when using this configuration compared to a more dorsiflexed position (ankle joint at 85 degrees) that would increase the contribution of the gastrocnemii due to a rightward shift in the force-length relationship of the contractile elements. Further, our analysis of MTU mechanical properties does not account for potential age-related differences in knee extensor muscle strength and patellar tendon stiffness. One might argue that this may limit the validity of our findings, as next to the plantar flexors also the knee extensors contribute to leg stiffness and generated total muscular power during maximal DJs (Arampatzis et al., 2001a). However, since the age-related degeneration in muscle strength and tendon stiffness seems to be higher for the plantar flexors as for the knee extensors (see review McCrum et al., 2018a), we do not expect that this drawback significantly affects our main findings. Please note that in contrast to previously reported age ranges for middle-aged adults (typically 40–60 years; e.g., Süptitz et al., 2013; Saul et al., 2015) the current study included participants until the age of 67 years. However, the aim of the current study was basically to examine if matching younger and middle-aged adults for TS muscle strength and tendon stiffness eliminates age-related differences in muscular power production during DJ rather than age-related differences in neuromotor function *per se*. Despite the fact that seven of our middle-aged adults were between 41 to 59 years, we observed functionally relevant (≥36%) differences for the main outcome parameters between the age groups, indicating that including a higher number of participants over the age of 59 years would have even strengthened the findings of the current study. Finally, a limitation of the current study is the relatively low number of participants in each group (*n* = 8 for the young; *n* = 8 for the middle-aged group) which reduces the potential for detecting statistical differences between age groups. Therefore we cannot exclude that a higher number of subjects might have led to significant differences in jumping height between young and middle-aged adults. However, this drawback has no effect on our observation that matching young and middle-aged adults for TS muscle strength and tendon stiffness does not eliminate age-related differences in mechanical power output during jumping.

## Conclusion

In conclusion, independent of task demand, matching younger and middle-aged adults for TS isometric muscle strength and tendon stiffness eliminates age-related differences in jumping performance but not in mechanical power production during maximal DJs. Leg stiffness during the support phase was found to be significantly associated with muscular power production during maximal DJs, with lower leg stiffness values in middle- compared to younger-aged adults. Thus, while jumping performance appears to be unaffected when leg extensor muscle strength and tendon stiffness are maintained, the reduced muscular power output during lower limb multi-joint tasks seen with aging may be due to age-related changes in motor task execution strategy rather than due to muscle weakness.

## Data Availability

The data supporting the conclusions of this manuscript will be made available by the authors, without undue reservation, to any qualified researcher.

## Ethics Statement

This study was carried out in accordance with the recommendations of the German Sport university’s human ethical commitee. The protocol was approved by the German Sport university’s human ethical commitee. All subjects gave written informed consent in accordance with the Declaration of Helsinki.

## Author Contributions

KK, SH, and MK conceived the work. SH, TA, and MK acquired the data. MK drafted the manuscript. All authors contributed to analysis and interpretation of the work, prepared the figures, contributed to final approval of the version to be published, and agreed to be accountable for the work.

## Conflict of Interest Statement

KK has equity in Protendon GmbH & Co. KG, whose device and software was used for the data collection, processing, and analysis in this study. The remaining authors declare that the research was conducted in the absence of any commercial or financial relationships that could be construed as a potential conflict of interest. The handling Editor declared a shared affiliation, though no other collaboration, with one of the authors CM.
